# Host Genetics and Environment Drive Divergent Responses of Two Resource Sharing Gall-Formers on Norway Spruce: A Common Garden Analysis

**DOI:** 10.1371/journal.pone.0142257

**Published:** 2015-11-10

**Authors:** E. Petter Axelsson, Glenn R. Iason, Riitta Julkunen-Tiitto, Thomas G. Whitham

**Affiliations:** 1 Department of Wildlife, Fish and Environmental Studies, Swedish University of Agricultural Sciences, Umeå, Sweden; 2 The James Hutton Institute, Craigiebuckler, Aberdeen AB15 8QH, Scotland; 3 Department of Biology, University of Eastern Finland, PO Box 111, Joensuu 80101, Finland; 4 Merriam-Powell Center for Environmental Research & Department of Biological Sciences, Northern Arizona University, Flagstaff, Arizona, United States of America; University of Arkansas, UNITED STATES

## Abstract

A central issue in the field of community genetics is the expectation that trait variation among genotypes play a defining role in structuring associated species and in forming community phenotypes. Quantifying the existence of such community phenotypes in two common garden environments also has important consequences for our understanding of gene-by-environment interactions at the community level. The existence of community phenotypes has not been evaluated in the crowns of boreal forest trees. In this study we address the influence of tree genetics on needle chemistry and genetic x environment interactions on two gall-inducing adelgid aphids (*Adelges* spp. and *Sacchiphantes* spp.) that share the same elongating bud/shoot niche. We examine the hypothesis that the canopies of different genotypes of Norway spruce (*Picea abies* L.) support different community phenotypes. Three patterns emerged. First, the two gallers show clear differences in their response to host genetics and environment. Whereas genetics significantly affected the abundance of *Adelges* spp. galls, *Sacchiphantes* spp. was predominately affected by the environment suggesting that the genetic influence is stronger in *Adelges* spp. Second, the among family variation in genetically controlled resistance was large, i.e. fullsib families differed as much as 10 fold in susceptibility towards *Adelges* spp. (0.57 to 6.2 galls/branch). Also, the distribution of chemical profiles was continuous, showing both overlap as well as examples of significant differences among fullsib families. Third, despite the predicted effects of host chemistry on galls, principal component analyses using 31 different phenolic substances showed only limited association with galls and a similarity test showed that trees with similar phenolic chemical characteristics, did not host more similar communities of gallers. Nonetheless, the large genetic variation in trait expression and clear differences in how community members respond to host genetics supports our hypothesis that the canopies of Norway spruce differ in their community phenotypes.

## Introduction

Responses to genetically determined trait expressions in a common host plant often vary among members of associated communities and with environmental conditions. Such responses would be consistent with the formation of an extended community phenotype. A recent review shows that community and ecosystem phenotypes are now recognized in diverse ecosystems around the world (e.g. tropical to temperate regions, marine to alpine systems), and can be found with taxonomically divergent plants such as grasses and forbs to trees [1]. Despite their widespread occurrence we know relatively little about how the genetics of coniferous trees may affect associated communities in boreal forests [1] but see studies on; understory vegetation [2, 3], endophytes [4] and mycorrhizae [5, 6]. In contrast, although the arthropod communities inhabiting the canopies of boreal trees can be quite diverse [7] little is currently known of the influence of host genetics on these communities.

Variability in genetically controlled traits can be large in boreal trees [8, 9] and as such could translate into effects on ecologically important processes such as species interactions. Genetic variability in resistance against pests and diseases may affect interactions with individual pest species [10–15]. For example, Björkman [11] studied the galling aphid *Adelges abietis* and was able to define different Norway spruce genotypes as resistant and susceptible. Resistant trees had both lower abundance of galls and a negative effect on aphid performance. Further, both Tjia et al. [15] and Björkman [11] found yet unexplored relationships between *Adelges abietis* and specific but unidentified individual phenolic substances in spruce needles. Together these studies confirm a high variability of genetically determined trait expressions within boreal tree populations and that selection for particular properties may change how traits are expressed in the population.

Genetically determined trait expressions may also affect whole communities of associated organisms as shown in other systems. For example, Whitham et al. [16] showed that the variation in arthropod community composition associated with the canopies of *Populus* trees was predominantly associated with genetically determined concentrations of condensed tannins in the leaves. Similar effects have been reported for the understory vegetation of a boreal tree [3] and soil microbial communities [2, 4–6, 17].

Furthermore, both plants and their associated organisms are highly responsive towards their environment and as such the interaction between genetics and environment (G x E) are of key importance for community and ecosystem phenotypes [11, 18–21]. Further, the different community members occurring on the same host may be considered as a biotic part of the environment that constitutes an additional driving or interacting factor. Thus, if species sharing the same resource differ in their response towards host genetics and/or interactions between host genetics and the environment (G x E) this would suggest a genetic effect on interaction strength, which varies with environment, potentially having cascading effects on community composition.

In the formation of genotype-specific communities, interactions among community members are of key importance [19, 22–26]. Herbivorous insects for example, interact strongly both with their host and other species of herbivores through facilitation and competition, and resource exploitation and manipulation of host resources [27]. The interspecific interactions occurring on genotypes are strong determinants of the distribution and abundance of species [28, 29]. For example, Khudr et al. [28] showed how plant genetics influenced the outcome of competition so that the population increase of the pea aphid (*Acyrthosiphon pisum*) depended on both host plant genotype and the identity of competing species. Population growth varied as much as four fold on different genotypes when competing with the vetch aphid (*Megoura viciae*). However, when comparing population growth on the same genotypes when competing with the green peach aphid (*Myzus persicae*) these differences switched direction so that the formally `better`host genotype now had reduced population growth rates of the aphid. Given such effects it is evident that the influence of interactions among community members can also be genotype-dependent and contribute to the patterns of community composition and community phenotypes [24].

In this study we use a common garden approach to evaluate the potential for community phenotypes in Norway spruce (*Picea abies*). We studied on different scales, the influence of host genetics and environment on gall formation by a small community of adelgids (*Sacchiphantes* spp. and *Adelges* spp.) utilizing the same resource for gall formation/reproduction. On a regional scale we investigate the relative influence of spruce genetics and environment on gall formation by the two adelgids in two common gardens in the boreal zone of northern Sweden. Through this approach we ask 1) if the two gall-inducing adelgid aphids (*Adelges* spp. and *Sacchiphantes* spp.) respond consistently to variation in host genetics (G), environment (E) and the G x E interaction? Further, on a local scale we address the relationship between host genetics, host chemotype and gall formation on spruce trees. Here we ask 2) if there is variation in genetically determined traits such as phenolics, putative plant defenses of Norway spruce, and/or resistance, and 3) if variation in phenolics can help explain patterns of gall formation? This allows us to investigate the existence of and mechanisms underlying community phenotypes of crown invertebrates. Our findings are significant as they extend the concept of community phenotype to a system that to the best of our knowledge has not been studied before (e.g. the canopy gall-forming arthropods of boreal trees) and show how G x E interactions can influence community phenotypes just as environmental interactions can influence traditional phenotypes of morphology and other traits.

## Material and Methods

**Fig 1:**
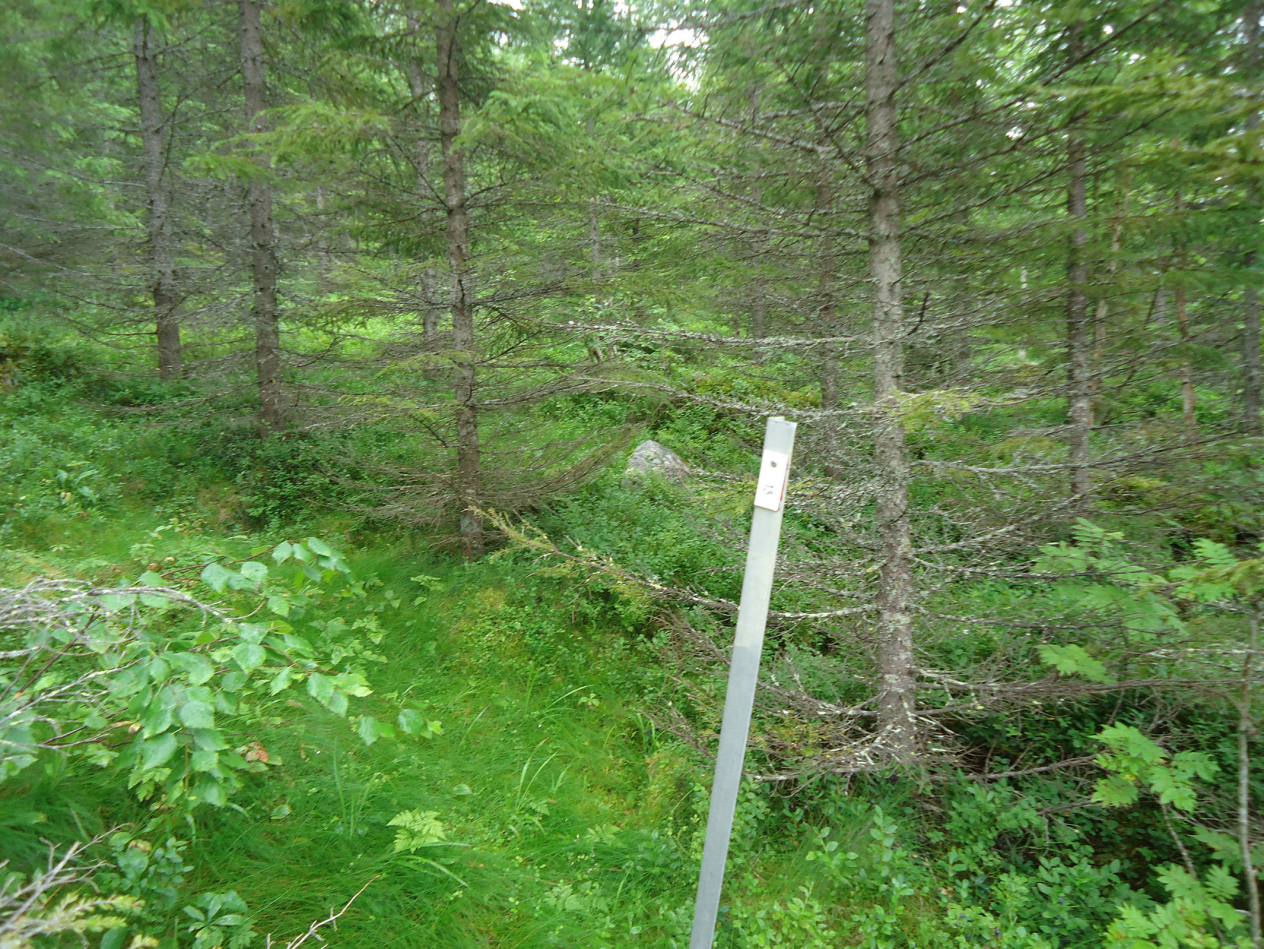
Picture from the coastal site Bjursjön showing the 30 year old Norway spruce trees in rows separated at 2.2m and trees growing at 1.7m intervals within rows.

Two common gardens set up and managed by the Forest Research Institute of Sweden (Skogforsk) were used in this study (ID nr S23F8320392 and S23F8320393). Permission to use both sites was granted by Skogforsk and the studies did not involve endangered or protected species. The gardens are located at Bjursjön approximately 80 km north of Umeå in the county of Västerbotten, Sweden (64°20'N 20°21"E, altitude 240m) and Myrträsk 40 km west of the city of Lycksele (64°29'N 17°54"E, altitude 490m). The two sites are spatially separated by 130 km and differ in abiotic conditions. Bjursjön is a coastal site, located 40 km from the Gulf of Bothnia with relatively warm summers and mild winters. Myrträsk is situated 150 km from the coast and has a cooler climate with harsher winters. The location of the sites results in a vegetation growing season (the number of days >5°C) of about 160 days at the coastal and 140 days at the inland region. Also, the large differences in altitude (240m and 490m in Bjursjön and Myrträsk, respectively) add to these climatic differences which are also manifested in plant growth. At a survey ten years after establishment the plants was about 50% higher in the coastal low altitude site compared with the inland high altitude site. At establishment in 1983, 87 spruce full-sibs, originating from crosses between mother and father trees from different regions throughout Sweden, were planted in a complete randomized design at both sites. Rows were planted 2.2 m from each other and within rows plants was separated by 1.7m from each other. At the time of survey the trees in Bjursjön were roughly 8–10 cm in diameter at breast height although both smaller and larger threes was observed (Fig 1).

For our field survey we randomly selected 17 fullsib families. These full-sib families represent controlled crosses between mothers and fathers originating from latitudes between 60.33 and 64.28 and altitudes between 260m and 550m a.s.l. (Table 1). Gall abundance was surveyed in August of 2013 (when trees were 30 years old) at both sites on the ten lowest green branches on each tree by counting the total number of galls induced on the tree by the two insect species. This included galls infecting the tree over several seasons and serves as a good metric for resistance. Gall abundance in this context reflects the plants ability to avoid infection (antixenosis) which are known to correlate with pest performance (antibiosis) in Adelges [11] and other gall forming arthropods [30, 31]. Trees that were dead were excluded from surveys and further analyses as they were judged to be unrepresentative and families were included only if there was a minimum of 4 trees representing each fullsib family at each site. In total we recorded data on gall occurrence on 77 individuals from the common garden at Bjursjön, and gall occurrence and chemistry on 79 individuals from the common garden at Myrträsk. Data on gall occurrence can be accessed through the supporting information (S1 File).

**Table 1:** The latitude and altitude of the parents of the 17 full-sib families surveyed for gall abundance in these studies.

Family		Mother			Father	
	Latitude		Altitude	Latitude		Altitude
5	63.80		260	60.52		325
6	62.62		460	60.52		325
10	60.42		330	60.52		400
11	63.80		260	60.52		400
17	63.80		260	61.22		550
19	60.98		480	61.22		550
22	60.42		330	61.53		460
29	63.80		260	61.03		380
46	61.58		480	61.47		490
48	61.17		360	61.47		490
69	60.87		495	60.87		465
77	60.62		330	60.48		310
88	60.33		300	63.68		240
100	60.33		300	64.28		410
104	64.28		410	64.28		410
110	64.28		410	60.52		420
123	64.28		325	60.42		330

Chemical composition was addressed by sampling needles from the most peripheral current-year-shoot of the lowest green branch without gall infection on each tree. The samples were air dried at room temperature (~22°C) before extraction and analyses with high-performance liquid chromatography (HPLC). Approximately 10 mg of material was used for analyses of needle phenolics. The sample was homogenized for 20 s at 5500 rpm with Precellys 24 (Bertin technologies, France) in 600 μl of cold 100% methanol. After 15 min of incubation in an ice bath the sample vial was centrifuged for 3 min at 16 100 g and the supernatant was collected. Re-homogenizing was repeated with 5 min standing on ice 3 more times. This repeated procedure gives more than 95% recovery of all phenolics found in the samples. Methanol from combined supernatants was dried in a vacuum centrifuge (Eppendorf 270 concentrator, Germany), redissolved in 600 μl of 50% methanol and analysed with HPLC using a water:methanol gradient according Julkunen-Tiitto and Sorsa [32]. The HPLC-instrument (Agilent, Series 1100, Germany) containing a Diode Array Detector (DAD) (G1315B) was combined with HP ChemStation Software. The HPLC column used was ZORBAX C-18 Rapid Resolution column (particle size 3.5 μm, dimensions 4.6× 75 mm, Agilent Technologies, Germany). Autoinjection volume was 20 μl, and injector and oven temperatures were 30 and 23°C, respectively. Standards used for quantification of phenolic compounds at 270 nm (for acetophenon/picein, catechin, lignans, phenolic acids and stilbenes) and 320 nm (for flavonoids) were as follows: picein (Sigma, Germany) for acetophenones; kaempferol 3-glucoside (Sigma-Aldrich, Germany) for kaempferol and its derivatives; isorhamnetin 3-glucoside (Sigma-Aldrich, Germany) for isorhamnetin; quercitrin 3-glucoside (Extrasynthese, France) for quercetin derivates; apigenin 7-glucoside (Sigma-Aldrich, Germany) for apigenin derivatives; luteolin 7-glucoside (Sigma-Aldrich, Germany) for luteolin derivatives; naringenin -7-glucoside (Sigma-Aldrich, Germany) for naringenin derivatives; chlorogenic acid (Sigma, Germany) for phenolic acids; piceatannol (Sigma-Aldrich, Germany) for stilbenes; (+)-catechin (Sigma, Germany) for catechins, and salicin (Sigma, Germany) for lignans. Further identification of the soluble non-tannin phenolic compounds was done according to Taulavuori et al. [33] using UHPLC-DAD (Model 1200 Agilent Technologies)-JETSTREAM/QTOFMS (Model 6340 Agilent Technologies) equipped with a 2.1 x 60 mm, 1.7μm C18 column (Agilent technologies). The soluble condensed tannins were analyzed from HPLC samples with acid-butanol assay for proantocyanidins. Condensed tannins extracted from needles of *P*. *abies* were used as standards in quantification. Data on phytochemistry can be accessed through the supporting information (S1 File).

Norway spruce in the region are known to be infected by a small community of galling adelgids from two different genera; *Sacchipantes* spp. (*S*. *viridis* and *S*. *abietis*) and *Adelges* spp. (*A*. *laricis* and *A*. *tardus*). The two taxa cause morphologically divergent and thus easily distinguishable galls. Galls of *Sacchipantes* spp. are a `pineapple gall`adelgid and are characterized by a dark green color with the edges of `scales`shifting towards red or brown with the infected shoot typically extending beyond the gall but do eventually die. Galls of *Adelges* spp. are a `strawberry gall`adelgid characterized by a yellowish color, with the infected shoot not extending beyond the gall, instead the gall may or may not have a tuft of needles extending out from its end [34]. Due to these differences in appearance, galls of *Sacchiphantes* spp. and *Adelges* spp. can be distinguished on previous year’s shoots even though they have lost their color. Although the potential interactions between these two species are poorly understood the use of this same resource, e.g., manipulation of emerging buds in spring to form their galls provide a strong interface for interactions between *Sacchiphantes* spp. and *Adelges* spp. The mutually exclusive pattern of this utilization, e.g. infection of one gall prevents infection by the other and results from other systems suggest that interactions between different galling species can be substantial. Compson et al. [35] used 14C labelling experiments to demonstrate that galls represent artificial sinks and that the presence of multiple galls can reduce individual performance. Still, observational evidence suggests that Sacchiphantes spp. occur on more vigorous shoots whereas Adelges spp. less vigorously growing shoots suggesting that they may partition the bud/shoot resource to minimize competition.

### Statistical analyses

The effects of site, genotype and their interaction on gall occurrence were tested using Residual Maximum Likelihood estimates calculated using Generalized Linear Modelling in Genstat version 16 [36]. Site was considered a fixed effect and was tested using an F test, whereas Genotype and its interaction with Site was considered a sub-sample of the entire population and treated as random variables, the effects of which were tested using changes in residual deviance compared with a Chi-squared distribution, following their inclusion in the model. Preceding the analyses residual plots were used to check the assumptions of normal distribution and equal variances. Both *Adelges* spp. and *Sacchiphantes* spp. revealed skewed patterns and consequently; data was Log + 1 transformed before use in analyses. We also explored interspecific relations by pair-wise correlation analyses on the abundance of the two genera.

The relation between family, chemical phenotypes and gall occurrence was explored with a combination of different multivariate statistics (discriminant- and principal component analyses), and ANOVAs and generalized linear models using the statistical software JMP 11 pro. First, discriminant analyses were performed to see if genotypes differed in chemical profiles. This was done using a canonical plot with 95% confidence ellipses where non overlapping ellipses denote significant differences. Second, the multivariate chemical data, e.g., the 31 different chemical substances, were simplified into 3 uncorrelated components through principal component analyses. These were later used in generalized linear models to explore the relationships between chemistry and gall occurrence of the two species separately.

We also explored the relationship between community similarity and environmental (chemical) similarity to test a component of the genetic similarity rule [37] in which genetic similarity is correlated with phytochemistry, which in turn is correlated with community composition. Community similarity was expressed as Bray-Curtis dissimilarity and the environmental similarity was expressed as Euclidean distances [38]. The data on galls were transformed (log[x+1]) prior to the calculation of the matrices to improve linearity and was zero adjusted to account for zero scores. The two matrices of community (Bray-Curtis distance) and environmental similarity (Euclidean distance) were then used in Mantel test to determine if more similar environments in terms of needle chemistry hosted more similar communities of galls. Dissimilarity-based methods such as Mantel tests can be applied to any number of species, from one to a whole community. The test of the phytochemical similarity rule was done in R (v 3.1.2) using the ecodist library [38].

## Results

### Galler distributions

**Fig 2:**
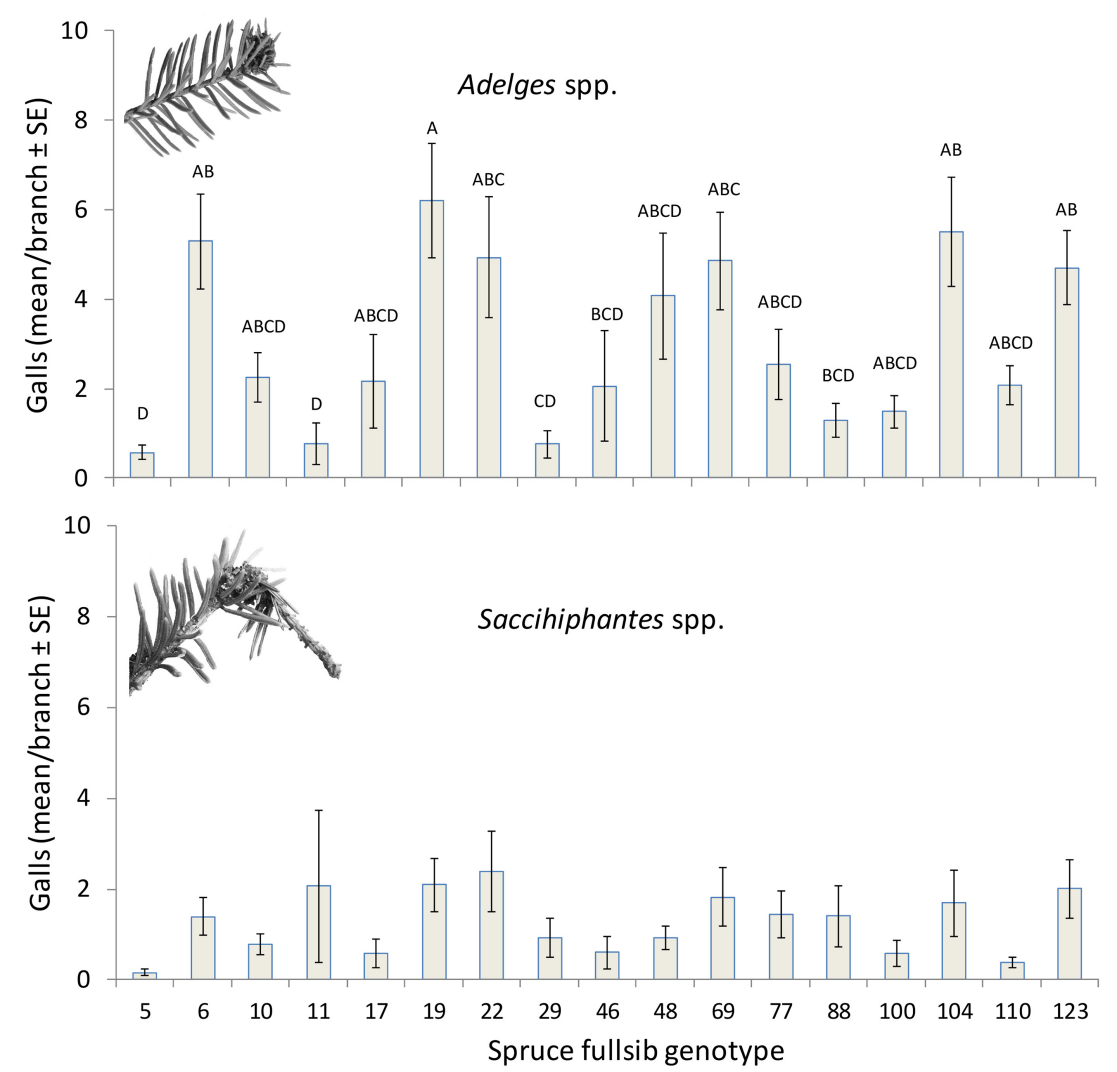
Gall abundance (mean/branch ± SE) of *Adelges* spp. and *Saccihiphantes* spp. on different spruce full sib families growing in common gardens. Bars with different letters are significantly different (Tukeys HSD, P < 0.05).

The two galls clearly differed in their occurrence and in general, *Adelges* spp. galls were more common than *Sacchiphantes* spp. galls (Fig 2). The two species also differed in their general response to spruce family and environment (Table 2). The genetic origin of Spruce trees had a strong and significant effect on the abundance of *Adelges* spp. gall infections (*P* < 0.001) so that the infection in some cases differed as much as 10 fold among families (Fig 2). Site (E) and the G x E interaction had no significant effect on *Adelges* spp. infection (Table 2). In contrast, *Sacchiphantes* spp. was not affected by genotype and responded significantly only to environment (Table 2, *P* < 0.05). Mean abundance of *Sacchiphantes* spp. was ~ 50% higher in the costal site Bjursjön than in the inland site Myrträsk (1.53 ± 0.20 and 0.99 ± 0.23, respectively). The G x E interaction was not significant for *Sacchiphantes* spp. (Table 2). Pair-wise correlation analyses showed a generally positive relation between abundance of galls of *Sacchiphantes* spp. and *Adelges* spp. (r = 0.3687, *P* < 0.0001) but that this relation was only significant in Bjursjön and not in Myrträsk (r = 0.5970, *P* < 0.0001 and r = 0.1525, *P* = 0.1798, respectively).

**Table 2:** Results from Generalized Linear Modelling showing the effects of site, genotype and their interaction on gall formation of *Adelges* spp. and *Sacchiphantes* spp. adelgids on spruce trees.

Dependent variable	Explanatory variables	Test statistic	df	Probability
*Adelges* (log X+1)	Site (Fixed)	F = 1.95	1,16	NS
	Genotype (Random)	Χ^2^ = 29.16	1	<0.001
	Genotype x Site (Random)	Χ^2^ = 1.90	1	NS
*Sacchiphantes* (log X+1)	Site (Fixed)	F = 5.40	1,16	<0.05
	Genotype (Random)	Χ^2^ = 2.39	1	NS
	Genotype x Site (Random)	Χ^2^ = 2.55	1	NS

### Phytochemistry

**Fig 3:**
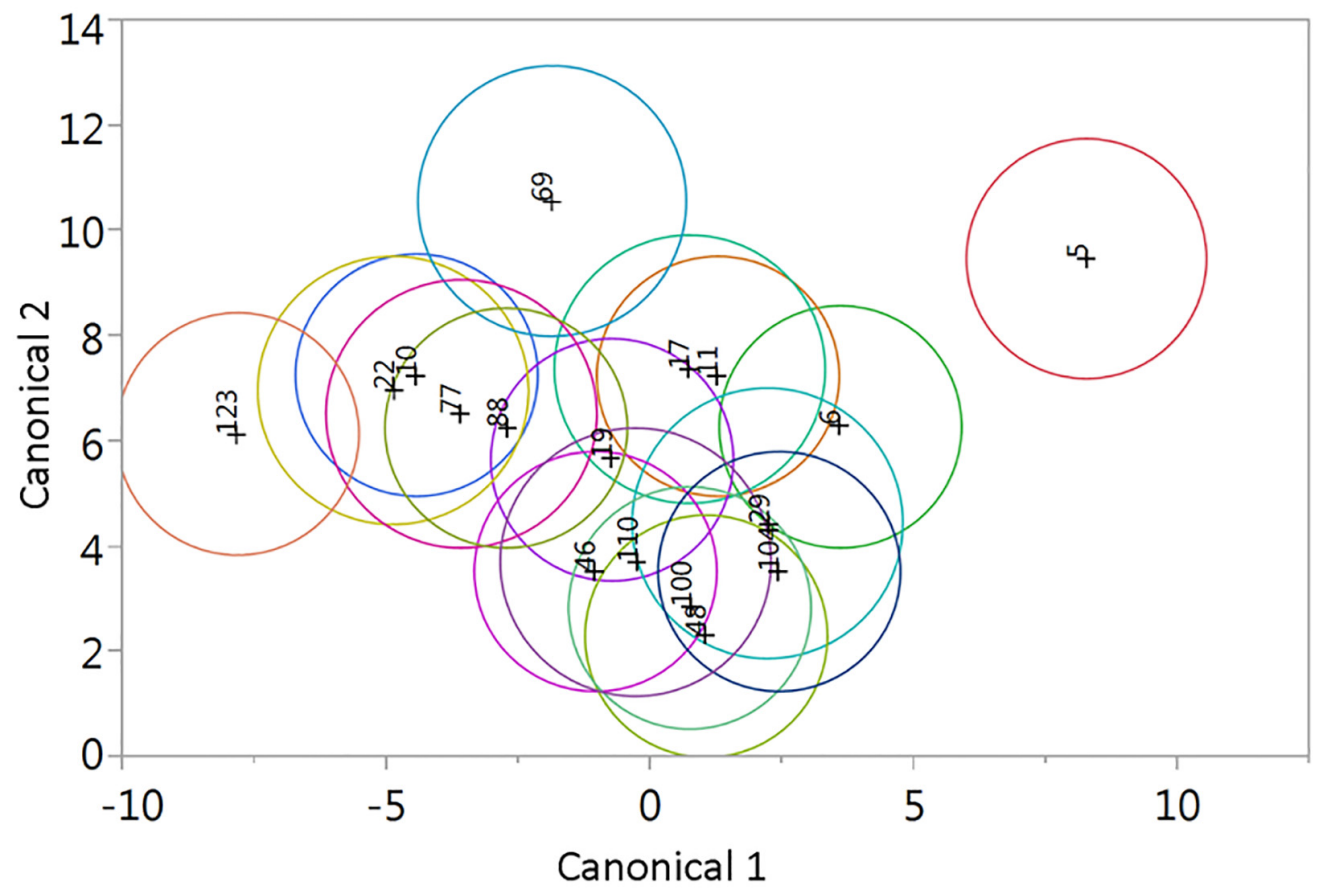
Two dimensional canonical plots showing the distribution of chemical profiles of needles from different spruce full-sib families growing in a common garden. Non-overlapping confidence ellipses (95%) around canonical means illustrate that the variation in chemistry within groups are smaller than the variation among groups and explain the significant difference found in discriminant analyses (Wilks’ λ F = 1.8240, P < 0.0001).

The discriminant analyses on needle chemistry revealed large variability and also a continuous overlap of chemical profiles of different spruce families (Fig 3). The exception from this general overlap was family 5 which was completely separated from all other families. Despite the continuous overlap in distribution, significant differences among spruce families were quite common (Fig 3). For example, the most peripheral families 5, 69 and 123 revealed clear separation and there were many examples of non-overlapping confidence ellipses among the more centrally distributed chemical profiles (Fig 3).

Through the principal component analyses on the 31 different chemical substances we established 3 principal components (PC) explaining ~45% of the variation (22.2, 11.8 and 11.2% for PC 1, PC 2 and PC 3, respectively, Table 3). The use of these three components in generalized linear models revealed that PC 2 had a significant relationship with *Sacchiphantes* spp. galls and that PC 3 had a significant relationship with gall infection by *Adelges* spp. galls (Table 4). Based on eigenvector scores it is shown that PC 2 is predominately characterized by apigenin and apigenin-glycoside and PC 3 mainly by naringenin derivatives and luteolin (Table 3). Naringenin derivatives was expressed only in three samples in total, all in family 6 suggesting that their influence at a population level may be limited. Apigenin derivatives occurred in all samples and regression analyses indicate a weak negative relationship between total concentration of apigenin deratives and infection by *Sacchiphantes* spp. galls (*P* = 0.0625, R^2^ = 0.044). Similarly, the relationship between luteolin derative and infection by *Adelges* spp. is weak but close to significant (P = 0.0543, R^2^ = 0.047).

**Table 3:** The loading of each of 31 chemical substances along the 3 principal components (PC) subsequently used in generalized linear models to explain the relationship between needle chemistry in spruce and gall infection by *Sacchiphantes* spp. and *Adelges* spp. Together these 3 components explained 45% of the variation. Bold letters indicate compounds with a high loading on the principal components that show significant relationship with gall infection of *Sacchiphantes* spp. (PC 2) and *Adelges* spp (PC 3).

Eigenvectors	PC 1	PC 2	PC 3
Picein	0.24726	-0.00127	-0.18439
Cinnamic acid derivative	0.00675	-0.22571	0.04230
4-hydroxy acetophenone	-0.04179	-0.04439	0.11392
(+)-catechin	0.04377	0.02656	-0.16367
Piceatannol-glucoside	0.22142	0.08186	-0.19902
Cedrusin-glucoside	-0.19826	-0.02973	0.19850
Cedrusin derivative	-0.07962	-0.01780	-0.03289
Piceatannol	0.09148	0.26130	-0.19374
Isorhapontin	-0.16191	0.07510	0.00114
Secoisolariciresinol	0.19196	-0.19121	0.12071
Naringenin-glycoside	-0.15663	0.26688	**-0.30965**
Naringenin derivative 1	-0.13065	0.25931	**-0.30222**
Hyperin	0.02571	0.24881	0.20534
Quercetin 3-glucoside	-0.04414	-0.13851	0.21443
Resveratrol	-0.24109	0.22840	-0.01838
Apigenin-glycoside	0.13595	**0.31310**	0.08334
Kaempferol 3-glucoside	0.22089	0.06869	0.21307
Isorhamnetin	0.12366	0.22730	0.22028
Kaempferol 3-rhamnoside	0.28049	0.17550	0.00879
Myricetin	0.26196	0.18777	0.07050
Naringenin derivative 2	-0.16014	0.26242	**-0.30388**
Luteolin derivative	-0.07808	0.24387	**0.35418**
Apigenin derivative 1	-0.04510	0.12180	0.07286
Monocoumaroyl-isoquercitrin	0.20812	0.20640	0.02708
Apigenin	-0.11395	**0.30401**	0.26731
Monocoumaroyl-astragalin	0.13131	0.14079	0.16911
Apigenin derivative 2	0.31574	0.12642	0.04632
Dicoumaroyl astragalin 1	-0.09413	-0.00975	0.19325
Dicoumaroyl astragalin 2	-0.33292	0.09545	0.05823
Dicoumaroyl astragalin 3	-0.30707	0.10805	0.10129
Soluble tannins	0.12589	-0.03025	-0.18331
	0.6494	-0.00243	-0.34375

**Table 4:** Likelihood Ratio (L-R) Chi-Square statistic and significance values generated from generalized linear model analyses on the relation between chemical profiles of spruce needles (expressed as 3 principal components) and galling of *Adelges* spp. and *Sacchiphantes* spp. on spruce trees growing in a common garden.

	*Adelges*		*Sacchiphantes*	
Source	L-R Chi^2^	*P*	L-R Chi^2^	*P*
PC 1	2.2098	0.1371	0.3620	0.5474
PC 2	0.0467	0.8289	5.3622	0.0206
PC 3	6.4152	0.0113	0.1302	0.7183

### Test of phytochemical and gall community similarity

The test of phytochemical and gall community similarity revealed no pattern. A Mantel test was insignificant suggesting that trees with more similar phytochemistry did not host more similar communities of gallers (*P* = 0.739, Mantel r = -0.0398).

## Discussion

### Genetic and environmental effects on gallers and community phenotypes

Our findings show that these two galling adelgids, which share the same shoot niche, exhibit divergent responses towards variation in host genetics and environment. Whereas the infection by *Adelges* spp. was strongly influenced by genetics, which was consistent over the two study environments, the response of *Sacchiphantes* spp. was predominantly explained by environment and less influenced by genetics. As in other traditional genetics-based traits such as morphology and phenology, gene x environment can affect their expression. Although the observed G x E effects on the two gallers were not strong, our contrasting results for two community members clearly suggest that interactions between G x E help define the community phenotypes of galling arthropods in Norway spruce.

The divergent response shown here may result from the combined effects of genetics and the environment, and interactions among community member of this small community of galling adelgids. Although *Sacchipantes* spp. and *Adelges* spp. galls had the potential to compete for the same shoot resources for gall formation and reproduction, we found a positive relationship between the abundance of the two genera on the same tree. This suggests that the interaction might not be so competitive or that the two galls have similar preferences for individual trees. Within trees they might still have different preferences or be subjected to resource partitioning such that overlap on the same shoots or branches are minimal. Positive relations between different herbivores are common [39–41] although not uniform [40]. Significant correlations between resistances are indicative of diffuse selection such that multiple herbivores may collectively exert selection for resistance [41]. In our study this positive relationship was only significant in one of the sites (r = 0.5970), which again points to the environmental importance for genetically based species interactions and community composition. Community phenotypes can be driven by both plant traits such as phytochemistry, morphology and phenology as well as genetic-based interactions among community members acting independently or in concert. Similar to our finding of an environment-dependent variable relationship between *Sacchipantes* spp. and *Adelges* spp., Compson, Larson, Zinkgraf & Whitham [35] showed how competition between two gallers of the same aphid species (*Pemphigus betae*) on *Populus angustifolia* was greater on susceptible than resistant tree genotypes. The interspecific interactions occurring on individual plant genotypes are strong determinants of the distribution and abundance of species [28] and the composition of their community, which are therefore shaped by host genetics [18, 42, 43]. Divergent responses towards genetically determined trait expressions in the host or food plant are common [19, 26, 44, 45] and suggest that interspecific interactions may vary in strength or even direction depending on genotype [28]. Clear differences in how different community members respond to the genetics of their common host as found in this study, is evidence for the existence of community phenotypes. Although the genetic effect was clearly significant we need to acknowledge that the magnitude of this effect may depend on spatial scale. In this study the full-sib families originate from a spatial scale that is larger than the garden replication, which may influence how the magnitude of the effect is interpreted [46]. Further support for the hypothesis of community phenotypes of Norway spruce are findings that tree soil interactions of coniferous boreal trees can influence the composition of ectomycorrhizal communities [5, 6, 17].

### Phytochemistry and resistance

In response to our second question our results confirm that the studied spruce population contains large genetic variability, in this case in resistance and needle chemistry, with potential ecological importance. We show that *Adelges* gall infections differed as much as 10 fold in different spruce families suggesting a clear genetic influence over resistance. Abundance of galls is often correlated with pest performance [11, 30, 31] and thus confirm a good estimate of resistance. The genetic influence is in line with previous studies demonstrating the genetic control over resistance against adelgids [10, 11, 13]. Our study also revealed genetically determined variability in needle chemistry. As expected there was a continuous overlap among different spruce families in chemical profiles, but still there existed clear and significant differences among some of the families. Chemotypic variability in trait expressions are ecologically important as they may translate to influence important ecological associations [3, 12, 16] or ecosystem processes [47, 48]. Genetically determined plant traits such as leaf or needle chemistry often have a large influence on associated communities [3, 16, 49]. For example, Whitham et al. [16] showed that approx. 55% of the variation in arthropod community composition associated with the canopies of *Populus* trees could be explained by genetically determined concentrations of condensed tannins in the leaves.

Our third question relates to mechanistic links between host phenotypes (phenolics) and gall formation. Despite predictions that host chemistry should affect associated organisms our analysis on needle phenolics and gall infections show only a limited connection. Earlier studies have however established connections between host chemistry and infection by *Adelges* galls [11, 15, 50]. Similar to Tjia et al. [15] Björkman [11] matched one as yet unidentified individual phenolic compound which, in contrast to total phenols, tended to increase in susceptible trees. These studies provide an important validation of the approach used here addressing a spectrum of different substances. Still, despite our analyses identifying 31 specific substances we were unable to establish convincing connections between phenolic chemistry and gall infection. Through the use of three principal components as descriptors of needle phenolic chemistry, we show limited relationships with gall infection. Relations were only confirmed for the principal components explaining least (~10%) of total variation among families. Similarly, associated with a lack of significant relationships between phenolic chemistry, our test of similarity revealed no relation between chemical environment and galling communities. In other studies where defense chemistry is associated with resistance, plants that are chemically similar also support more similar communities of arthropods [37].

Overall, a poor connection with needle phenolic chemistry but still clear genetic control suggest that adelgid galls may respond to other genetically determined traits in Norway spruce that were not addressed here, i.e. the important among-family differences might be related to other traits such as other groups of defensive chemicals or to physical traits [50] or, as has been found in other galling species, to ontogeny [51] or sink-source relationships [35]. The relative importance of other plant traits and phytochemistry have been debated in recent years [52, 53] and a lack of general pattern suggests that the attributes of the considered herbivore may determine the plant traits of importance [52]. The reason for poor links between chemistry and infection in our study may potentially also stem from the fact that needle chemistry in this study relates to current concentrations whereas gall infection is representative of the infections accumulated on the trees over several years or that the gall-inducing stem-mothers are responding to the characteristics of the buds and not of the needles. Still, consistency among years in a genetically determined qualitative character (such as needle chemistry), suggests that sampling of galls across multiple years are in fact reflecting their response to consistent differences among trees.

### Extended considerations

The existence of community phenotypes as repeatedly demonstrated in various studies suggests pronounced genetic effects on communities and whole ecosystems. For example, if genotypes all with their own more or less unique community are mixed in the landscape, biodiversity of associated communities would increase [54, 55]. Wimp et al. [55] showed that plant genetic diversity accounted for nearly 60% of the variation in arthropod diversity in a populus hybrid system. The genetics and trait expression of forest trees are currently modified in boreal forests of Sweden and elsewhere, e.g. approximately 75% of forest regeneration in Sweden is done through planting with plant material originating from seed orchards and selected for their production potential [56]. Due to the weight of evidence supporting the existence of community and ecosystem phenotypes in boreal trees [2, 3, 5, 6, 17], regeneration strategies that select for a subset of plant families (e.g., high productivity) are likely to have unintended consequences on their associated communities and overall biodiversity [1] just as genetic modification of specific traits can have unintended consequences for dependent communities [57]. The full extent of the genetic influence in boreal forests is currently unknown and needs to be quantified to understand their full environmental significance. Similarly, studying the effects of interactions between genes and the environment (G x E) is essential to understand the influence of community and ecosystem phenotypes. For example, Roder et al. [7] studied the arthropod communities of Norway spruce and showed that total arthropod richness decreased linearly with increased elevation, which reflected declining temperatures, and predicted that further climate warming will promote overall species richness. Understanding how the genetics of boreal forest trees interact with environment to affect community and ecosystem phenotypes would prove valuable in mitigation of the effects of ongoing climate change.

## Conclusions

We conclude that the two adelgids studied here show divergent responses toward the genetics of their shared host. This represents indicative support of the emerging evidence that boreal trees host community and ecosystem phenotypes just as shown in other forest systems around the world. Given such effects, management strategies that alter the genetic structure of tree populations may have yet unappreciated effects on associated communities and whole ecosystems. Given that boreal forests are experiencing changes in the genetics of forest trees and a simultaneous change in climate the interaction between the genetics of foundation trees and climate is essential for the understanding of the effects of anthropogenic changes in boreal forests.

## Supporting Information

S1 File Excel document with data on gall occurrence and phytochemistry. (XLSX)
